# Do Children with Autism Spectrum Disorders (ASD) Have Deep Learning Ability? An Exploratory Research in Inclusive Play

**DOI:** 10.3390/jintelligence13110135

**Published:** 2025-10-27

**Authors:** Yanrong Zhu, Xueyun Su

**Affiliations:** 1 Department of Early Childhood Education, East China Normal University, Shanghai 200062, China; 52284104006@stu.ecnu.edu.cn; 2 Normal School of Early Childhood Education, Lanzhou City University, Lanzhou 730070, China; 3 Research Institute of Care and Education of Infants and Young Children, Shanghai 200241, China

**Keywords:** deep learning ability, children with autism spectrum disorders, inclusive play

## Abstract

Deep learning ability is critical for children’s development, yet little research has been conducted on preschool children with autism spectrum disorders (ASD). This study investigated the deep learning ability of children with ASD in inclusive play, which involved 8 children with ASD and 13 children with typical development (TD) (5–7 years of age) from four public kindergartens in China. An assessment scale for children’s deep learning ability was developed, and children were observed during inclusive play (IP) and solitary play (SP) in natural settings. A total of 40 play cases (10 were IP and 30 were SP) were collected. Key findings indicated that (1) children with ASD had a moderate level of deep learning ability during play, the emotional experience scored the highest while the interpersonal interaction scored the lowest. (2) The score of deep learning ability in children with ASD in SP was higher than that in IP. (3) Monthly per capita household income, father’s occupation, siblings, and primary playmates significantly influenced the deep learning ability of children with ASD. This study provided new insights into deep learning during play for children with ASD and offered an empirical basis for future inclusive education.

## 1. Introduction

Deep learning is a learning process in which children, with the support of teachers, actively engage in challenging problem-solving situations. Through hands-on activities and interpersonal interactions, they transfer and apply their prior knowledge, ultimately solving real-world problems and constructing new cognitive structures (Guo 2016; Wang 2021; Wu 2019). Constructivist theory provides an epistemological foundation for deep learning. Rather than involving the mechanical transmission of objective knowledge directly instilled into children’s minds, deep learning emphasizes the active construction of individual experience through contextual and cultural interactions in the learning process (X. Chen 2019). It represents a state of children’s active learning driven by internal motivation. Deep learning also emphasizes the transfer and application of experience, highlighting its constructive nature and capacity for transfer. This aligns with the core principles of constructivist learning theory, which emphasizes learners’ autonomous construction of knowledge, integration of perspectives, and application of prior experiences to solve problems (L. Huang 2022). At the same time, children’s prior experiences constitute the field of vision through which they formulate questions, observe problems, and analyze them during deep learning (Shi 2001). Deep learning ability is both a prerequisite for children to engage in deep learning and a capacity that develops progressively throughout the process. It refers to a comprehensive capability that extends beyond cognitive capacities to encompass six dimensions: emotional experience, problem awareness, transfer and application, problem solving, interpersonal interaction, and cognitive quality.

Since the 1980s, research related to deep learning has flourished, yielding increasingly rich scholarly outcomes. However, while much of the research on deep learning has focused on children with typical development (TD), little attention has been paid to children with autism spectrum disorders (ASD) in early childhood. The existing research has primarily focused on supporting the entire process of screening, diagnosis, intervention, and development in children with ASD (Abdelwahab et al. 2024; Frangi et al. 2018), or on applying the principles of artificial intelligence and deep learning neural networks to enhance ASD detection accuracy (Rubio-Martín et al. 2024), diagnostic scrutiny (Feng and Xu 2023), and multiple classification of brain MRI (Nogay and Adeli 2024).

Autism spectrum disorders (ASD) is a complex neurodevelopmental disorder characterized by deficits in social interaction, verbal communication, and perception, as well as restricted interests, stereotyped behaviors, and frequent intellectual impairments (Chen et al. 2024). Existing research on children with ASD predominantly focuses on their multiple functional deficits, including those in language, social interaction, and executive functions. In terms of behavior, children with ASD often exhibit unconscious behaviors such as purposelessness and impaired perceptual abilities, which are characterized by stereotyped and repetitive patterns of behavior and a reduced ability to perceive potential dangers (American Psychiatric Association 2014; Imbiriba et al. 2023). They may also experience difficulties with executive functioning skills, which relate to behavioral inhibition or regulation, planning and organization (Fleury et al. 2014; Schaefer Whitby and Mancil 2009). Stereotypical behaviors further exacerbate their difficulties in adapting to the social environment (Shi et al. 2023). In terms of problem-solving, children with ASD demonstrate detail-focused thinking rather than global thinking and experience difficulty maintaining focus and planning the steps involved in problem-solving (Happé and Frith 2006). They also face challenges in memory, attention, self-monitoring, and abstract thinking (Fleury et al. 2014; Schaefer Whitby and Mancil 2009). These symptoms can impose significant physical and psychological stress on children with ASD during interpersonal interactions. In the long term, children with ASD not only struggle to integrate into the community but also face significant challenges in academic performance and developmental progress (Hu 2023; Koegel et al. 1992). Although children with ASD exhibit deficits in multiple domains of functioning, some individuals demonstrate enhanced cognitive processing, sustained attention, visuospatial abilities, creativity, and other notable strengths. Firstly, many children with ASD are visual learners and demonstrate distinct strengths in visuospatial abilities (Jolliffe and BaronCohen 1997). The most common manifestation of this strength is the ability to rapidly locate a target object within a complex visual scene, which is commonly referred to as superior visual search performance (Happé and Frith 2006). Secondly, specific creative abilities can be found among children with ASD (Hetzroni et al. 2019). They demonstrated greater originality and stronger verbally creative abilities compared to their age-matched peers with TD (Kasirer and Mashal 2014; Kasirer et al. 2020). In addition, children with ASD often exhibit enhanced attention and demonstrate a pronounced strength in mechanical memory (Ai and Wang 2022). Overall, most research reflect a deficit perspective, and few studies provide insights from a more strength-based perspective.

This study aimed to investigate whether children with ASD demonstrate deep learning ability in autonomous play contexts, where they were free to choose their activities independently. Accordingly, this research adopted a whole-child perspective and sought to investigate the current state and existing challenges related to the deep learning ability of children with ASD in inclusive play, with the goal of supporting the further development of their deep learning ability.

## 2. Deep Learning Ability During Play Among Children with Autism Spectrum Disorders

### 2.1. Deep Learning and Inclusive Play

Preschool children’s deep learning doesn’t focus on advanced learning materials (Wang and Liu 2020), as it is closely linked to play, during which children often raise questions that introduce new themes and extend the play (Lu et al. 2019). Play also provides a context for problem-solving, transfer, and application, enabling children to deepen their learning through inquiry and interaction. Some researchers argued that children acquire culture through play, gradually learning to use tools to solve problems, and exploring and mastering social interaction, language expression, and creativity necessary for participation in future adult societies (Bruner et al. 1976). Thus, play significantly influences the depth of learning.

In this study, inclusive play encompassed two meanings. On the one hand, it referred to supporting children with ASD in participating in play and social interactions with TD peers (Casey 2005; Sobel 2018), guided by the principles of equality, acceptance, and inclusion. On the other hand, inclusive play served as the research context, providing a balanced focus on the deep learning ability of children with ASD and children with TD during play. It aimed to compare and analyze these abilities in order to explore effective learning strategies. Therefore, this study served as an important approach to achieving a win-win outcome for both children with ASD and children with TD, aiming to create opportunities for social interaction and inclusion (Fernelius and Christensen 2017).

Inclusive play is particularly important for children with ASD, as it not only provides them with opportunities to engage in social interactions, but also provides them to learn from others and to recognize differences between themselves and their peers (Petrie and Poland 1998). In addition, playing together can be critical in changing the perception of children with TD toward disability and enhancing recognition of children with ASD (Yantzi et al. 2010). More importantly, by feeling and experiencing the physical and mental pleasure brought about by inclusive play, children with ASD gain the freedom and dignity of being a “subject person” in their own right (Li and Liu 2022). This lays the foundation for deep learning in children with ASD.

### 2.2. Play Among Children with Autism Spectrum Disorders

Children with ASD can gain increased opportunities to learn through play, which lays the foundation for promoting the development of their cognitive abilities such as attention, perception, memory, language, and thinking, as well as fostering self-regulation skills (Grandland 2014). However, children with ASD generally exhibit low levels and a more limited variety of play, making it difficult for them to achieve the same physical and mental development outcomes as children with TD (Hong 2022).

Stereotypic behaviors often accompany children with ASD, who frequently spend prolonged periods manipulating an object in a repetitive and restricted manner without a clear goal, such as repeatedly hitting or continuously spinning it without pause (Zhu and Zhou 2014). The selection of toys is not guided by a preference for specific object characteristics, and children seldom use toys in an appropriate manner or engage in complex play with them (Tilton and Ottinger 1964). In addition, children with ASD are constrained by verbal communication impairments, which limit their ability to engage in social interaction during play and impede peer interactions, thereby contributing to stereotypical behaviors and diminished imaginative capacity (Wolfberg et al. 2014). This also impairs their representational abilities (Baron-Cohen et al. 1985), as they struggle to use—particularly a symbolic substitute—to represent another, such as using shells to represent cats or letters to represent stones (Hughes 1998). Consequently, children with ASD are less likely to engage in symbolic or pretend play (Sigman and Sena 1993) and exhibit the lowest level compared to children with intellectual disabilities and those with TD (Zhou and Fang 2004). In addition to verbal communication deficits, this might also stem from a lack of interest in play (Lewis and Boucher 1988). and difficulties in perceiving the function of toys correctly (Zhou and Fang 2004).

Although most children with ASD engage in play at a simple and repetitive level, play remains rich in opportunities for rehabilitation and learning. For example, enhancing the enjoyment and motivation of children with ASD during play can improve their sensorimotor play skills (Mao 2011). Play also exerts a significant positive influence on the social communication abilities of children with ASD (Wen et al. 2024). Therefore, integrating speech-language therapy into play can effective enhance the verbal communication skills of children with ASD (Xiong et al. 2022), thereby laying the foundation for interpersonal interaction. In addition, during symbolic play therapy, children with ASD can fully engage in pretend and imitative behaviors (S. Chen 2010) and learn to think from different perspectives, which contributes to significant improvements in their theory of mind abilities (Xiao et al. 2014).

### 2.3. Influences on Learning in Children with Autism Spectrum Disorders

Numerous factors influence the learning and development of children with ASD. Firstly, children with ASD tend to pay less attention to the social environment and people, while focusing more on non-social stimuli. They exhibit a cognitive processing style characterized by attention to minute details or constituent elements, often at the expense of holistic processing. This pattern reduces opportunities for social interaction and learning, thereby hindering learning through imitation (Su 2014). Secondly, IQ is an important indicator of intelligence, and IQ scores below 70–75 are generally considered to indicate significant intellectual functioning deficits. According to the DSM-5, autism spectrum disorders is often associated with intellectual developmental disorders (American Psychiatric Association 2014), which poses substantial challenges to children’s information processing.

Thirdly, children with ASD exhibit significant age-related improvements in group participation and number concept skills (Ando et al. 1980). Some studies have also shown that the main effect of age groups on cognitive function in children with ASD is not significant. The cognitive function scores across all age groups remain consistently low, indicating an extremely delayed developmental trajectory that shows minimal improvement with increasing age (Zhao et al. 2015). In addition, the overall developmental level of adaptive behaviors in children with ASD tends to decline with increasing age (Fenton et al. 2003; Zhao et al. 2015).

Furthermore, an active learning format enables children with ASD to learn more successfully (Shipova et al. 2025). While social anxiety is significantly negatively associated with academic performance in normal students, it is significantly positively associated with academic performance in the ASD group. Additionally, the interaction term between ASD and social anxiety is a significant predictor of academic performance (Zukerman et al. 2019). Weak memory leads to significant difficulties in recalling and re-recognizing repeatedly presented information (Zhou 2002). Compared to spoken material, children with ASD demonstrate a relative strength in observing and remembering visual stimuli such as graphics; therefore, instruction that incorporates drawings, video images, and similar visual supports is beneficial for their cognitive development (Lei 2004).

### 2.4. Research Questions

Investigating the current status and challenges of deep learning ability in children with ASD is both valuable and meaningful, as it establishes a foundation for supporting their development, informs the design of inclusive play, helps identify key developmental needs to be addressed during play. Therefore, the deep learning ability of children with ASD in inclusive play and solitary play were assessed. The research questions were:

1. Do children with ASD demonstrate deep learning ability during play?
2. Are there significant differences in deep learning abilities among children with ASD across gender and age? And are there significant differences in the deep learning abilities of children with ASD in different types of play?
3. What factors influence children’s deep learning ability?

## 3. Methods

### 3.1. Ethics

Prior to the study, approval was obtained from the Institutional Review Board of East China Normal University (IRB number HR2-0272-2025, date of approval 1 July 2025). Before conducting the survey, children and their parents were recruited through kindergarten parent-teacher conferences, parent-child activities, bulletin boards, and online platforms, and informed consent was obtained from all participants.

### 3.2. Participants

The study involved 21 children (5–7 years of age, IQ ≤ 50) from four public kindergartens in China, including 8 children with ASD (Gender: Females = 1, Males = 7; Age: Mean = 5.75, SD = 0.707) and 13 children with typical development (Gender: Females = 6, Males = 7; Age: Mean = 5.77, SD = 0.439). As shown in Table 1. Only one girl with ASD among the four kindergartens involved in this study. In addition, the IQs of the children with ASD in the four kindergartens were measured by qualified pediatricians in government-appointed hospitals, and the IQs of the eight children with ASD in the sample were ≤50 according to the official file; however, due to the characteristics of the children’s impairments and the limitations of the assessment tools, they may not fully reflect the children’s actual cognitive levels.

During play, children can freely choose play materials, themes, and peers based on their individual interests. They engage in autonomous play within familiar classroom settings or outdoor environment, utilizing materials routinely available in the kindergarten. The kindergartens provided a wide variety of manipulative and low-structured play materials for children, including those set up or placed in the garden, such as 0.5 m × 0.5 m sandboxes filled with sensory sand, molds, small pots; spray bottles; plastic pieces of various shapes and colors; plastic plates; modeling clay in multiple colors; plastic tubes of different lengths and connectors; skipping ropes, tires, rollers; sponge mats, ladders; sand pits, and cardboard boxes, among others.

Assessments were conducted in naturalistic play settings, incorporating on-site coding and real-time evaluation of children’s deep learning ability. The assessment began when the child initiated play and ended upon completion. Play duration varied, depending on the child’s developmental stage and capacity for deep learning. Researchers assessed children’s deep learning ability during both inclusive play (IP) and solitary play (SP). Inclusive play includes at least one child with ASD and one child with TD forming a playgroup and engaging in a shared play theme or with common play materials. Given that children with ASD often lack joint attention, it is not required for them and their TD peers to cooperate toward the same play goals; instances of parallel but co-located play are permitted within inclusive play. However, in 60% of the cases collected for this study, children participating in inclusive play demonstrated verbal or behavioral interactions, either spontaneously or under teacher guidance. The four Chinese kindergartens each conducted 30 min of inclusive play daily at a fixed time. Implementation of inclusive play varied due to differences in classroom settings. In kindergartens with special education classes, regular class teachers guide 3 to 5 children with TD into the special education classes each day to participate in inclusive play, with each child participating at least once per semester. In kindergartens with inclusive education classes, all children engaged in play in open outdoor environment, during which they could independently and spontaneously choose their playmates. Solitary play has frequently been characterized in existing research as a distinct and self-contained form of play, wherein the child engages in independent activities without regard to the actions of others, plays alone with toys that differ from those used by nearby children, and shows no initiative to interact or physically approach peers within speaking distance (Parten 1932). However, solitary play in the present study diverges from this traditional definition by incorporating a focus on different types of children, including children with ASD and children with TD. Specifically, solitary play is conceptualized in relation to inclusive play, emphasizing whether children with ASD and those with ASD participate in play activities together. Specifically, solitary play is conducted individually by children of different types, in which children with ASD or TD select their preferred toys and engage in play either independently or in playgroups with peers of the same developmental type or with a teacher.

### 3.3. Instrument of Observation

“Assessment Scale for Children’s Deep Learning Ability during Play” was developed for this study based on Wang’s interpretation of the logical framework for children’s deep learning (Wang and Liu 2020) and Fredericks’ three-dimensional input theory of cognition, emotion and behavior (Fredricks et al. 2004). The scale included 6 primary indicators and 23 secondary indicators. Each secondary indicator was rated on a five-point scale based on children’s behavioral performance, ranging from high to low, with a total of 115 assessment items. The assessment scale indicators and reliability coefficients for each subfactor are presented in Table 2.

The structure and elements of the assessment were revised and validated through Delphi expert consultation and surveys conducted by eleven experts independently. Kendall’s coefficient of concordance was applied to test the Inter-rater reliability (IRR) of the 11 experts, and the results showed that Kendall’s W was 0.749 (X^2^ = 49.407, *p* < 0.001), indicating high consistency. The Kaiser-Meyer-Olkin (KMO) value (0.834) and Bartlett’s test of sphericity (X^2^ = 233.014, *p* < 0.001) indicated that the data were suitable for factor analysis. The results of the factor analysis revealed six extracted factors with eigenvalues greater than 1, accounting for a cumulative variance of 78.292% after rotation, as presented in Table 3 and Table 4. Cronbach’s alpha for the scale was 0.895, and Cronbach’s alpha for every dimension was higher than 0.8, indicating high internal consistency.

In this study, the Varimax rotation with Kaiser Normalization was used to find the correspondence between the factors and the research items. It can be seen that all the research items correspond to a communality value higher than 0.5, which means that there was a strong correlation between the research items and the factors, and that these factors can be used to extract information effectively.

### 3.4. Research Procedure

**Figure 1:**
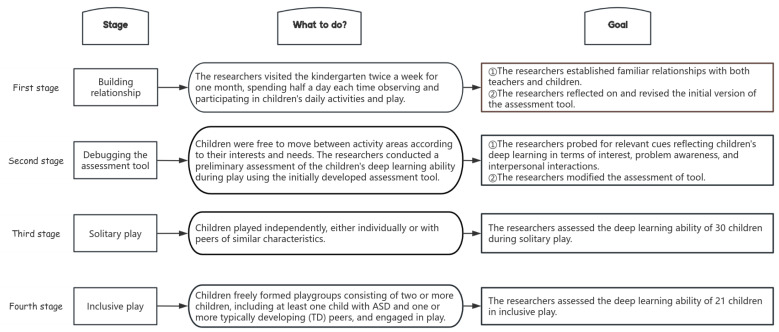
Research process.

In the process of conducting the study, the research team and the early childhood teachers held fortnightly online meetings to clarify the assessment ideas and the application of the assessment tools, as well as to maintain a unified assessment standard. Moreover, new problems encountered during the assessment process were negotiated to reach a consensus solution. The research process consisted of four distinct stages, as illustrated in Figure 1.

In this study, a total of 40 cases of children’s play were observed and collected, including 10 inclusive play cases and 30 solitary play cases (23 for children with ASD and 7 for children with TD). Among the 10 IP cases, one case involved two children with ASD and one child with TD, while the remaining 9 cases involved one child with ASD and one child with TD, a total of 21 assessments were conducted in the IP (11 for children with ASD and 10 for children with TD). Therefore, a total of 51 assessments of children’s deep learning abilities (34 for children with ASD and 17 for children with TD) were included. Notably, the number of assessments collected likely exceeded the number of participating children, as there was no one-to-one correspondence between assessments and participants. For example, a child with ASD might engage in multiple solitary play sessions at different times and with different toys, yielding multiple assessment records. The number of play cases and assessments is presented in Table 5.

### 3.5. Data Analysis

SPSS 27.0 was used to analyze the results of the assessments of 51 children’s deep learning abilities. First, descriptive statistics were used to analyze the current status of deep learning ability in children with ASD during play, as well as their ability in inclusive and solitary play. Second, the Shapiro–Wilk normality test was conducted to determine whether the results showed a normal distribution, providing a reference for deciding how to perform variance analysis. Third, the Mann–Whitney U test for two independent samples was used to verify significant differences in deep learning abilities among children with ASD based on different ages, genders, and types of play. Finally, simple linear regression was conducted to show which demographic variables affect the deep learning ability during play among children with ASD.

## 4. Results

### 4.1. Do Children with Autism Spectrum Disorders Demonstrate Deep Learning Ability?

The descriptive statistical analyses presented in Table 6 were conducted to investigate whether children with ASD were capable of deep learning in play.

Descriptive statistics showed that the average score of deep learning ability of children with ASD in play was 84.62 (SD = 17.741), indicating that children with ASD were capable of deep learning during play. Among the six dimensions, the emotional experience (M = 17.74, SD = 3.306) scored the highest, while interpersonal interaction (M = 9.29, SD = 3.958) scored the lowest.

In addition, descriptive statistical analyses of the deep learning ability demonstrated by children with ASD in inclusive play and solitary play is shown in Table 7, showing that the mean score of deep learning ability of children with ASD in solitary play (M = 88.57, SD = 18.771) is higher than the score in inclusive play (M = 76.36, SD = 12.355). The ordering of the scores on the six dimensions remained consistent.

### 4.2. Are There Differences Among Different Ages in the Deep Learning Abilities of Children with Autism Spectrum Disorders?

The Kruskal–Wallis test was carried out and showed that deep learning ability (5-year-olds: *n* = 8, 6-year-olds: *n* = 19, 7-year-olds: *n* = 7, H = 6.343, df = 2, *p* = 0.042 < 0.05), transfer and application (H = 7.117, df = 2, *p* = 0.028 < 0.05) and cognitive quality (H = 6.139, df = 2, *p* = 0.046 < 0.05) of different ages of children with ASD showed significant differences; the performance of 6-year-olds was significantly higher than that of the other two age groups.

The data for age 7 was set as the reference variable in a simple linear regression to predict the impact of age on children’s deep learning ability (Adjusted R^2^ = 0.74, F = 2.311, *p* = 0.116 > 0.05); however, the regression equation was not significant. The six dimensions were similarly not significantly affected.

### 4.3. Are There Differences Between Different Genders in the Deep Learning Abilities of Children with Autism Spectrum Disorders?

Results from Mann–Whitney U test showed that deep learning ability (Males: *n* = 31, Females: *n* = 3, U = 1.500, *p* = 0.006 < 0.01), emotional experience (U = 7.000, *p* = 0.011 < 0.05), transfer and application (U = 5.500, *p* = 0.010 < 0.05), problem solving (U = 4.000, *p* = 0.009 < 0.01), interpersonal interaction (U = 11.500, *p* = 0.032 < 0.05) and cognitive quality (U = 14.000, *p* = 0.048 < 0.05) of different genders of children with ASD showed significant differences.

After virtualizing females’ data by using males’ data as reference values, the simple linear regression results in Table 8 indicated that males’ deep learning ability was significantly higher than that of females (Adjusted R^2^ = −0.316, B = −35.774, *p* < 0.001). Significant differences were observed across all six dimensions.

### 4.4. Are There Differences Between Solitary Play and Inclusive Play in the Deep Learning Abilities of Children with Autism Spectrum Disorders?

First, a Shapiro–Wilk normality test showed that the data were non-normally distributed to some extent. Therefore, a non-parametric Mann–Whitney U-test in Table 9 and Table 10 showed that deep learning ability, problem solving and cognitive quality of children with ASD in the solitary play were significantly higher than their performance in the inclusive play.

Finally, the data of IP was virtualized by using the data of SP as reference values. The simple linear regression presented in Table 11 indicated that children’s cognitive quality in SP was significantly higher than that of IP. Deep learning and other dimensions were not significant.

### 4.5. Which Demographic Variables Influence Deep Learning Abilities During Play in Children with Autism Spectrum Disorders?

#### 4.5.1. Monthly per Capita Household Income

Using the monthly per capita household income of CNY 10,000 and above as the reference group, simple linear regression analysis presented in Table 12 revealed that children with a monthly per capita household income of CNY 10,000 or above exhibited significantly higher levels of transfer and application compared to those in households earning between CNY 2000 and CNY 5000 or between CNY 5000 and CNY 10,000. No significant differences were found for deep learning overall or for the other dimensions.

#### 4.5.2. Primary Playmates

Simple linear regressions were conducted to examine the influence of children’s primary playmates, such as parents, grandparents, siblings, community children, teachers, and peers in kindergartens or rehabilitation institutions, on their deep learning ability and the six dimensions. Peers in kindergartens or rehabilitation institutions were used as the reference group.

The results presented in Table 13 showed that children whose primary playmates were peers in kindergartens or rehabilitation institutions exhibited significantly higher deep learning ability compared to those whose primary playmates were parents, grandparents, or siblings. Among the six dimensions, emotional experience was significantly higher than that of children whose primary playmates were parents and siblings; problem awareness was significantly higher than that of children whose primary playmates were parents; transfer and application was significantly higher than that of children whose primary playmates were parents, grandparents, siblings and teachers; problem solving was significantly higher than that of children whose primary playmates were parents and siblings; interpersonal interaction was significantly higher than that of children whose primary playmates were parents and siblings; cognitive quality was significantly higher than that of children whose primary playmates were parents, grandparents, siblings and teachers.

#### 4.5.3. Siblings

The Mann–Whitney U test was conducted, revealing that children with ASD who do not have siblings exhibited lower deep learning ability (with siblings: *n* = 25, without siblings: *n* = 9, U = 42.000, *p* = 0.006 < 0.01), transfer and application (U = 46.000, *p* = 0.007 < 0.01), problem solving (U = 47.500, *p* = 0.011 < 0.05), interpersonal interaction (U = 47.500, *p* = 0.011 < 0.05), and cognitive quality (U = 36.000, *p* = 0.003 < 0.01) compared to those who have siblings.

Using data from children without siblings as the reference group, simple linear regression analysis indicated that children with ASD who have siblings exhibited significantly higher levels of deep learning ability (Adjusted R^2^ = 0.213, B = 19.276, *p* = 0.004 < 0.01), transfer and application (Adjusted R^2^ = 0.219, B = 4.160, *p* = 0.003 < 0.01), problem solving (Adjusted R^2^ = 0.151, B = 3.858, *p* = 0.013 < 0.05), interpersonal interaction (Adjusted R^2^ = 0.167, B = 3.876, *p* = 0.009 < 0.01), cognitive quality (Adjusted R^2^ = 0.243, B = 4.862, *p* = 0.002 < 0.01), compared to those without siblings.

#### 4.5.4. Parental Occupation

Lu Xueyi classified Chinese social strata into five levels based on occupational status and access to organizational, economic, and cultural resources (Lu 2002). Following this framework, parental occupations were categorized into five levels: Level 1: Workers, peasants, and unemployed individuals living in poverty with limited job security; Level 2: Self-employed service providers and laborers; Level 3: Junior professional and technical staff, small business owners, clerical staff, self-employed entrepreneurs, middle-and senior-level technicians; Level 4: Middle-level managers and executives in large- and medium-sized enterprises; Level 5: Senior executives and top managerial personnel in large enterprises, as well as senior professionals from large private companies.

Simple linear regressions were conducted to examine the influence of parental occupation on children’s deep learning ability and the six dimensions. Mothers’ occupations at Level 4 were used as the reference group, the results showed no significant differences in deep learning ability or any of the six dimensions. Subsequently, fathers’ occupations at Level 3 were set as the reference group, and the results are presented in Table 14.

The results showed that children whose fathers’ occupations were at Level 3 exhibited significantly lower deep learning ability and performance across all six dimensions compared to children whose fathers’ occupations were at Level 5.

## 5. Discussion

### 5.1. Children with Autism Spectrum Disorders Demonstrated Deep Learning Ability

Children with ASD showed a moderate level of deep learning ability during play, and emotional experience played a significant role in facilitating this process. It is widely acknowledged in established research that children with ASD experience difficulties in emotional expression and understanding, including challenges in recognizing and expressing their emotions. However, this does not imply an inability to generate or perceive positive emotional experiences. The results showed that 88.24% of children with ASD demonstrated interest in materials or social partners during play and exhibited noticeable positive emotional changes; 91.18% were able to engage in play proactively and spontaneously. These findings reflect the motivation of children with ASD to explore and actively solve problems. Because these experiences were driven by positive emotions, they promoted sustained engagement in play and helped prevent surface-level processing (Wang and Liu 2020). Therefore, teachers should recognize the deep learning ability of children with ASD during play, effectively leverage their benefits in proactive emotional experiences and visual learning, and create inclusive play scenarios featuring sensory-stimulating problem-solving tasks to foster positive emotions and encourage active exploration.

At the same time, it was evident that interpersonal interaction posed a challenge to deep learning in children with ASD. Nevertheless, children with ASD demonstrated a desire to express thoughts, needs, and problems, and were able to make attempts and adjust their interactive behaviors in response to others’ suggestions. However, 58.82% of children with ASD lacked the awareness and ability to initiate or respond to interpersonal interactions, and only 35.29% were able to sustain two or more consecutive interaction turns through gestures, vocalizations, sign language, facial expressions, body movements, or speech. These findings were consistent with established research indicating that children with ASD generally experience difficulties in social interaction and in acquiring interaction skills, including challenges in engaging in reciprocal dialogue, initiating or maintaining interpersonal relationships (Gu 2018), developing prosocial behaviors such as turn-taking, cooperation, and sharing, and identifying appropriate solutions when confronting peer conflicts. The more interactive the learning experience, the greater the depth of learning (Chi and Wylie 2014). Deeper and more meaningful formal learning is more likely to occur when interactions take place among children, teachers, and materials (Anderson 2003). Therefore, it is essential for educators to promote social interactions between children with ASD and their peers or adults, and to support them in acquiring and applying interpersonal communication skills (Yao et al. 2024).

In addition, the results of the study showed that children with ASD across different age groups exhibited significant differences in deep learning, transfer application, and cognitive quality, with 6-year-olds performing significantly better than those in the other two age groups. As children grow older, they accumulate more experience, which can support children with ASD in transferring and applying their prior experience to solve problems in similar contexts. However, 5-year-old children with ASD exhibited a small annual decline in IQ from baseline to year 8 (Girard et al. 2023). This finding may partially explain why 7-year-olds demonstrated lower competence compared to those in the 6-year-old age group. While the regression analysis revealed no significant differences in age. This was consistent with existing research findings, and the relationship between children’s cognitive level and age was not statistically significant (Zhao et al. 2015). This suggested that differences in children’s deep learning ability should be understood in relation to multiple influencing factors, rather than attributed solely to age.

In terms of gender, boys demonstrated significantly higher levels of deep learning ability than girls. They were generally able to transfer their prior experiences to solve problems, whereas 20% of girls struggled to do so even with external support. Boys frequently engaged multiple senses, materials, and tools to extend simple observations into more in-depth investigations, while girls tended to rely more heavily on sensory input alone. This pattern suggests that boys may prefer concrete, intuitive strategies and are more likely to adopt direct, mechanical approaches when addressing real-world problems (Lai et al. 2015). Furthermore, individuals’ prior experience influences the strategies they employ in problem solving to some extent (Wang and Zhang 2015), 33.33% of girls and 79.53% of boys employed tool manipulation, material interaction, or the application of prior knowledge and experience to solve problems. This pattern indicates that boys may prefer concrete, intuitive strategies and are more likely to adopt direct, mechanical approaches when addressing real-world challenges (Lai et al. 2015). In addition, the only girl’s primary playmates were her parents; she had no siblings, and her father’s occupation was classified at Level 3. These factors were found to be significantly lower compared to those in other dimensions in subsequent analyses. However, the sample size for girls was very small in this study, with only 3 out of 34 assessments (8.82%) involving girls, which may have compromised the reliability of the findings. Indeed, ASD is more prevalent in males than in females. To some extent, the gender ratio of the sample reflects the actual prevalence pattern among children with ASD.

### 5.2. Contrary to Common Recognition of Inclusive Play, Our Study Found a Paradoxical Result: Children with Autism Spectrum Disorders Demonstrated Stronger Deep Learning Abilities in Solitary Play than in Inclusive Play

Children with ASD demonstrated higher deep learning ability and cognitive quality in solitary play than in inclusive play. Excluding two non-verbal children, 85% of children with ASD used verbal self-expression to solve problems during solitary play, compared to 45.45% in inclusive play. Language is a critical medium through which children interpret the world and express emotions and needs, and expressive language development is strongly linked to future cognitive abilities, problem-solving skills, and deep learning (Conti-Ramsden and Durkin 2012). During solitary play with familiar teachers, children with ASD engaged in meaningful interactions—expressing thoughts, sharing opinions, or seeking help—contributing directly to problem solving. In contrast, during inclusive play, they were more likely to engage in parallel play near peers (Anderson et al. 2004), initiated fewer social interactions (Corbett et al. 2010), and responded inconsistently to overtures from TD peers (Wolfberg et al. 2014). Verbal attempts by children with ASD were sometimes ignored or rejected by TD peers (Chen et al. 2017; Wei et al. 2015). Thus, the lack of verbal interaction, especially with TD peers, significantly hindered problem solving, cognitive quality, and deep learning in inclusive settings. When children with ASD can clearly express their needs, frustration decreases and engagement in activities and social relationships improves. Augmentative and alternative communication (AAC) tools provide effective means for children with ASD to understand ongoing events and influence their environment, enabling challenging behaviors, such as tantrums and repetitive actions—to be recognized as expressions of emotional tension (Carr and Carlson 1993).

Children with ASD spend significantly more time in solo play than in other play contexts (W. Huang 2008). Even when opportunities for peer interaction are available, they tend to remain on the periphery of group play unless actively supported. research indicates that deep learning is enhanced when play is combined with teacher involvement through guided play, directed play, or direct instruction (Cheng et al. 2024). Children with ASD often exhibit delays in play behaviors and require more intentional and systematic teaching strategies (Thiemann-Bourque et al. 2011). In solitary play, teachers can provide timely and accurate responses through one-on-one interactions, facilitating deeper cognitive engagement. In contrast, during inclusive play, teachers must divide their attention between children with ASD and TD peers, limiting their capacity for dynamic, real-time adjustments in group interactions (Bahiraey 2010). This constraint, due to cognitive load and environmental distractions, reduces the likelihood of deep learning for children with ASD in inclusive settings. These findings suggest that children with ASD rely on familiar teachers during play, and that the teacher-child ratio significantly influences their learning outcomes. Therefore, improving the teacher-child ratio and assigning specialized support staff in inclusive classrooms are essential steps to better meet the needs of children with ASD. Moreover, teachers should intentionally scaffold the transition from teacher-dependent interactions to peer-mediated engagement, promoting both independence and social inclusion.

### 5.3. Monthly per Capita Household Income, Father’s Occupation, Siblings, and Primary Playmates of Children Have a Significant Influence on the Deep Learning Ability in Children with Autism Spectrum Disorders

The results of this study showed that children from households with a monthly per capita income of CNY 10,000 and above demonstrated significantly higher levels of knowledge transfer and application compared to those from families earning CNY 2000 to 5000 or CNY 5000 to 10,000. Additionally, when fathers were employed in occupational level 3, children’s deep learning ability and performance across all six dimensions were significantly lower than when fathers held occupational level 5 positions. Socioeconomic status (SES), as a key family environmental factor, is closely associated with the quality of life for families raising children with autism. Moreover, the influence of family capital on parent-child relationships is often subtle, yet it may affect children’s deep learning through mechanisms such as parental educational investment, time spent with the child, academic expectations, and pedagogical beliefs. Children from low-SES backgrounds are at greater risk for emotional and behavioral difficulties, exhibit poorer adaptability, experience more frequent negative emotions, and tend to lack essential social interaction skills—all of which pose significant challenges to their long-term development (Zhu and Zhang 2013). Furthermore, higher monthly per capita household income can facilitate broader developmental opportunities and richer experiential learning for children with autism.

Additionally, children with ASD who have siblings demonstrated significantly higher levels of deep learning, transfer and application, problem solving, interpersonal interaction, and cognitive quality compared to those without siblings. This finding contrasts with existing studies indicating that social-cognitive development in children with ASD may be further delayed when they co-reside with older neurotypical siblings, regardless of the presence of younger siblings (Martin and Ross 2005). In families where an older neurotypical sibling lives with a later-born child diagnosed with ASD, the older sibling—similar to parents—may compensate for the younger child’s delayed or atypical social development through everyday interactions. This modeling provides a developmental scaffold that supports observational learning and promotes deeper cognitive and social engagement in children with ASD.

Findings showed that children with ASD whose primary playmates were peers in kindergartens or rehabilitation institutions demonstrated significantly higher deep learning ability compared to those whose primary playmates were parents, grandparents, or siblings. Peer interaction is therefore a valuable resource in inclusive play and has a positive impact on the development of deep learning skills in children with ASD. The integrated playgroup model emphasizes stimulating the potential of children with ASD to initiate and engage in play within peer groups and in environments matched to their developmental level 1 (Mao 2011). These playgroups provide rich, structured opportunities for children with ASD in inclusive settings, fostering initiative and spontaneity through prompting, imitation, and peer modeling and guidance.

### 5.4. Limitations and Future Directions

This study was constrained by a small sample size, which limited both the number of inclusive play instances and the frequency of deep learning assessments conducted within inclusive play. As a result, the discrepancy analysis of deep learning abilities in children with ASD across different age groups, play types, and other analytical categories may be compromised. Additionally, the sample included only one girl, reflecting the current enrollment—only one girl with ASD across the four participating kindergartens. This substantial gender imbalance may limit the reliability and generalizability of findings related to gender differences. In addition, autism spectrum disorders are a complex neurodevelopmental condition characterized by substantial individual variability among affected children. This heterogeneity increases the challenges inherent in conducting related research. At the same time, because the study was conducted in a natural setting, it ensured that children play in a natural and authentic manner. However, this ecological validity came at the cost of scientific rigor, as researchers were unable to control all relevant variables, which may compromise the internal validity of the findings. Last but not least, this study assessed children’s deep learning ability over a relatively short period, which may limit the reliability of the findings. The ability and behavioral performance of children with ASD in accumulating prior experience, problem solving, interpersonal interaction, and cognitive quality are unlikely to undergo significant change within a brief inclusive play.

The assessment of early learning and development in children with ASD should be an ongoing process; therefore, evaluating their deep learning ability within project-based, successive, and multiple inclusive play is essential. Future research should aim to assess the same child’s deep learning ability in both solitary and inclusive play, enabling paired-samples t-tests to compare differences across play types. This approach would enhance the scientific rigor of analyses and allow for more precise control of confounding variables. Furthermore, it is essential to examine whether specific factors, such as environmental determinants, activities outside kindergarten, types of developmental disorders, as well as teachers’ instructional language and teaching behaviors—serve as potential triggers for changes in deep learning. Finally, to strengthen the reliability and generalizability of findings, studies must also recruit larger samples of children engaged in inclusive play.

## 6. Conclusions

This study explores the deep learning ability of children with autism spectrum disorders (ASD) during inclusive play. The findings showed that children with ASD were capable of deep learning during play, and their deep learning ability reflects a complex structure. This highlights their potential to actively engage as competent learners, taking on meaningful roles in their own development—offering preliminary considerations that could inform the fostering of purposeful and profound learning experiences for children with ASD. Recognizing the value of play, particularly inclusive play, is critical to supporting the development of deep learning ability in children with ASD. In early childhood education, educators are encouraged to develop an understanding of how children with ASD engage in deep learning and systematically foster such skills through intentional and inclusive play practices.

## Figures and Tables

**Table 1:** Characteristics of participants (*n* = 21).

Kindergarten	Number	Name	Gender	Type of Children	Age	Verbal Language or Not	Participant in Which Play
Kindergarten A	1	TM1	Male	Children with ASD	7	Verbal language	IP and SP
	2	TM2	Male	Children with ASD	5	Verbal language	IP
	3	TM3	Male	Children with ASD	5	Verbal language	IP and SP
	4	TM5	Male	Children with ASD	6	Not	SP
	5	TF1	Female	Children with ASD	6	Verbal language	SP
	6	DF1	Female	Children with TD	6	Verbal language	IP
	7	DF2	Female	Children with TD	6	Verbal language	IP
	8	DM1	Male	Children with TD	6	Verbal language	IP
	9	DM3	Male	Children with TD	5	Verbal language	SP
	10	DM4	Male	Children with TD	5	Verbal language	SP
	11	DF6	Female	Children with TD	6	Verbal language	SP
	12	DF7	Female	Children with TD	6	Verbal language	SP
	13	DF8	Female	Children with TD	5	Verbal language	SP
Kindergarten B	14	TM7	Male	Children with ASD	5	Not	SP
	15	TM8	Male	Children with ASD	6	Verbal language	IP
	16	DF4	Female	Children with TD	6	Verbal language	IP
Kindergarten C	17	TM9	Male	Children with ASD	6	Verbal language	IP and SP
	18	DM2	Male	Children with TD	6	Verbal language	IP
	19	DM7	Male	Children with TD	6	Verbal language	IP
Kindergarten D	20	DM5	Male	Children with TD	6	Verbal language	SP
	21	DM6	Male	Children with TD	6	Verbal language	SP

Notes. IP = Inclusive play; SP = Solitary play. T = Children with ASD, D = Children with TD, M = Males, F = Females; TM = Males with ASD, TF = Females with ASD, DM = Males with typical development, DF = Females with typical development.

**Table 2:** Indicators of “Assessment Scale for Children’s Deep Learning Ability during Play” and Reliability for Each Subfactor.

Norm	Name				Cronbach’s Alpha
Level 1	1 Emotional experience				
Level 2	1.1 Interest	1.2 Initiative	1.3 Positive emotion experience	1.4 Willpower	0.914
Level 1	2 Problem awareness				
Level 2	2.1 Attention	2.2 Identify differences	2.3 In-depth exploration of problems	2.4 Problem representation	0.883
Level 1	3 Transfer and Application				
Level 2	3.1 Transfer	3.2 Application	3.3 Material selection	3.4 Hands-on operation	0.885
Level 1	4 Problem solving				
Level 2	4.1 Speed of analyzing and responding to problems	4.2 Strategies for implementing problem-solving	4.3 Problem-solving methods	4.4 Solve problem	0.859
Level 1	5 Interpersonal interaction				
Level 2	5.1 Awareness of interpersonal interaction	5.2 Ability of interpersonal interaction	5.3 Maintaining Interpersonal interaction		0.886
Level 1	6 Cognitive quality				
Level 2	6.1 Account	6.2 Causal relationship	6.3 Reflection	6.4 Creativity	0.861
Score	115				

Notes. Each Level 2 is worth 5 points, each Level 1 is the sum of the scores of the level 2 indicators, and the total score for this assessment scale is 115 points.

**Table 3:** Total Variance Explained.

Component	Initial Eigenvalues			Extraction Sums of Squared Loadings			Rotation Sums of Squared Loadings		
	Total	% of Variance	Cumulative %	Total	% of Variance	Cumulative %	Total	% of Variance	Cumulative %
1	8.949	38.907	38.907	8.949	38.907	38.907	3.374	14.671	14.671
	2.700	11.737	50.644	2.700	11.737	50.644	3.141	13.658	28.329
3	2.392	10.400	61.044	2.392	10.400	61.044	3.099	13.472	41.801
4	1.536	6.678	67.722	1.536	6.678	67.722	2.928	12.729	54.530
5	1.354	5.886	73.608	1.354	5.886	73.608	2.741	11.919	66.449
6	1.077	4.684	78.292	1.077	4.684	78.292	2.724	11.843	78.292
7	0.670	2.913	81.205						
8	0.559	2.429	83.634						
9	0.517	2.249	85.883						
10	0.454	1.976	87.859						
11	0.402	1.747	89.606						
12	0.358	1.558	91.164						
13	0.326	1.419	92.583						
14	0.319	1.388	93.971						
15	0.286	1.245	95.216						
16	0.226	0.983	96.199						
17	0.185	0.804	97.002						
18	0.161	0.700	97.702						
19	0.158	0.685	98.387						
20	0.134	0.583	98.970						
21	0.107	0.464	99.434						
22	0.082	0.357	99.790						
23	0.048	0.210	100.000						

Extraction Method: Principal Component Analysis.

**Table 4:** Factor Loadings and Communalities from the Exploratory Factor Analysis with Varimax Rotation.

Item	Factor 1	Factor 2	Factor 3	Factor 4	Factor 5	Factor 6	Communality
1.1 Interest	0.858	0.155	0.132	0.057	0.058	0.247	0.845
1.2 Initiative	0.789	0.134	0.154	0.215	0.132	0.246	0.788
1.3 Positive emotion experience	0.862	0.095	0.098	0.198	0.042	0.156	0.826
1.4 Willpower	0.883	0.001	0.040	0.008	0.031	0.130	0.799
2.1 Attention	0.091	0.843	0.178	0.203	−0.064	0.016	0.796
2.2 Identify differences	0.062	0.801	0.204	0.237	0.081	0.168	0.779
2.3 In-depth exploration of problems	0.170	0.739	0.144	0.251	0.039	0.180	0.693
2.4 Problem representation	0.058	0.799	0.081	0.240	0.253	0.099	0.780
3.1 Transfer	−0.033	0.287	0.179	0.826	0.195	0.125	0.851
3.2 Application	0.324	0.248	0.031	0.748	0.266	0.104	0.808
3.3 Material selection	0.210	0.211	0.134	0.772	0.138	0.081	0.729
3.4 Hands-on operation	0.068	0.336	0.296	0.749	−0.051	0.085	0.776
4.1 Speed of analyzing and responding to problems	0.213	0.008	0.100	0.203	0.282	0.789	0.799
4.2 Strategies for implementing problem-solving	0.190	0.276	0.362	0.056	0.348	0.666	0.812
4.3 Problem-solving methods	0.219	0.227	0.054	0.025	−0.001	0.826	0.785
4.4 Solve problem	0.271	0.055	0.260	0.113	0.052	0.718	0.675
5.1 Awareness of interpersonal interaction	0.031	0.060	0.064	0.140	0.895	0.067	0.833
5.2 Ability of interpersonal interaction	0.103	0.056	0.384	0.142	0.794	0.118	0.825
5.3 Maintaining Interpersonal interaction	0.085	0.098	0.261	0.133	0.815	0.242	0.825
6.1 Account	−0.008	0.139	0.786	0.089	0.217	0.271	0.765
6.2 Causal relationship	0.202	0.177	0.728	0.070	0.368	0.063	0.747
6.3 Reflection	0.114	0.151	0.805	0.160	0.047	0.122	0.726
6.4 Creativity	0.142	0.173	0.762	0.262	0.172	0.126	0.744

Rotation Method: Varimax with Kaiser Normalization.

**Table 5:** The number of play cases and assessments.

	Children with ASD	Children with TD	Total
Inclusive play (*n* = 10)	11 (2 + 9)	10 (1 + 9)	21
Solitary play (*n* = 30)	23	7	30
Total	34	17	51

**Table 6:** Survey of the deep learning ability of children with ASD during play (*n* = 34).

Dimensions	Mean	SD	Min	Max
Emotional experience	17.74	3.306	9	20
Problem awareness	15.12	2.422	8	19
Transfer and application	16.06	3.781	7	20
Problem solving	14.06	4.104	4	20
Interpersonal interaction	9.29	3.958	3	15
Cognitive quality	12.35	4.220	4	19
Deep learning ability	84.62	17.741	44	109

**Table 7:** Survey of the deep learning ability of children with ASD in inclusive play and solitary play.

Dimensions	Inclusive Play (*n* = 11)				Solitary Play (*n* = 23)			
	Mean	SD	Min	Max	Mean	SD	Min	Max
Emotional experience	17.00	3.098	11	20	18.09	3.410	9	20
Problem awareness	14.36	2.248	11	18	15.48	2.466	8	19
Transfer and application	14.82	2.822	11	20	16.65	4.086	7	20
Problem solving	12.55	3.616	4	18	14.78	4.199	4	20
Interpersonal interaction	8.09	2.625	5	12	9.87	4.393	3	15
Cognitive quality	9.55	3.142	5	15	13.70	4.050	4	19
Deep learning ability	76.36	12.355	57	97	88.57	18.771	44	109

**Table 8:** The survey of different genders influenced the deep learning ability during play among children with ASD.

Dependent Variable	B	SE	β	t	Sig.	VIF	R^2^	Adjusted R^2^	F
Deep learning ability	−35.774	8.869	−0.581	−4.033	0.000 ***	1.000	0.337	0.316	16.269
Emotional experience	−5.925	1.739	−0.516	−3.408	0.002 **	1.000	0.266	0.243	11.612
Problem awareness	−4.151	1.293	−0.493	−3.209	0.003 **	1.000	0.243	0.220	10.299
Transfer and application	−6.645	2.003	−0.506	−3.318	0.002 **	1.000	0.256	0.233	11.009
Problem solving	−8.473	2.027	−0.594	−4.181	0.000 ***	1.000	0.353	0.333	17.480
Interpersonal interaction	−5.075	2.259	−0.369	−2.247	0.032 *	1.000	0.136	0.109	5.048
Cognitive quality	−5.505	2.402	−0.376	−2.292	0.029 *	1.000	0.141	0.114	5.255

Notes. * *p* < 0.05, ** *p* < 0.01, *** *p* < 0.001.

**Table 9:** Mann–Whitney U test results for deep learning ability of children with ASD in different types of play.

	Emotional Experience	Problem Awareness	Transfer and Application	Problem Solving	Interpersonal Interaction	Cognitive Quality	Deep Learning Ability
Mann–Whitney U	89.000	81.500	82.000	70.500	86.000	50.500	69.000
Wilcoxon W	155.000	147.500	148.000	136.500	152.000	116.500	135.000
Z	−1.467	−1.684	−1.690	−2.076	−1.500	−2.809	−2.118
Asymp. sig. (2-tailed)	0.142	0.092	0.091	0.038 *	0.134	0.005 **	0.034 *

Notes. * *p* < 0.05, ** *p* < 0.01.

**Table 10:** Ranks for deep learning ability of children with ASD in different types of play.

Dimensions	Types of Play	Number of Cases	Rank Means	Ranks
Emotional experience	SP	23	19.13	440.00
	IP	11	14.09	155.00
Problem awareness	SP	23	19.46	447.50
	IP	11	13.41	147.50
Transfer and application	SP	23	19.43	447.00
	IP	11	13.45	148.00
Problem solving	SP	23	19.93	458.50
	IP	11	12.41	136.50
Interpersonal interaction	SP	23	19.26	443.00
	IP	11	13.82	152.00
Cognitive quality	SP	23	20.80	478.50
	IP	11	10.59	116.50
Deep learning ability	SP	23	20.00	460.00
	IP	11	12.27	135.00

**Table 11:** The survey of different types of play influenced the deep learning ability during play among children with ASD.

Dependent Variable	B	SE	β	t	Sig.	VIF	R^2^	Adjusted R^2^	F
Cognitive quality	−4.150	1.389	−0.467	−2.987	0.005 **	1.000	0.218	0.194	8.924

Notes. ** *p* < 0.01.

**Table 12:** The survey of monthly per capita household income influenced the deep learning ability during play among children with ASD.

Dependent Variable	Model	B	SE	β	t	Sig.	VIF	R^2^	Adjusted R^2^	F
Transfer and application	CNY 2000–5000	−4.526	3.581	−0.205	−1.264	0.216	1.022	0.200	0.148	3.870
	CNY 5000–10,000	−3.241	1.229	−0.428	−2.636	0.013 *	1.022			
	CNY 10,000 and above	0								

Notes. * *p* < 0.05.

**Table 13:** The survey of primary playmates influenced the deep learning ability during play among children with ASD.

Dependent Variable	Model	B	SE	β	t	Sig.	VIF	R^2^	Adjusted R^2^	F
Deep learning ability	Parents	−42.607	6.244	−0.785	−6.824	0.000 ***	1.134	0.661	0.615	14.157
	Grandparents	−21.857	8.325	−0.294	−2.625	0.014 *	1.076			
	Siblings	−21.857	4.333	−0.598	−5.045	0.000 ***	1.202			
	Teachers	−12.357	8.325	−0.166	−1.484	0.149	1.076			
Emotional experience	Parents	−5.500	1.555	−0.544	−3.536	0.001 **	1.134	0.395	0.311	4.729
	Grandparents	0.500	2.074	0.036	0.241	0.811	1.076			
	Siblings	−3.250	1.079	−0.477	−3.011	0.005 **	1.202			
	Teachers	−1.537 × 10^−15^	2.074	0.000	0.000	1.000	1.076			
Problem awareness	Parents	−4.071	1.242	−0.550	−3.279	0.003 **	1.134	0.281	0.182	2.837
	Grandparents	−0.571	1.656	−0.056	−0.345	0.732	1.076			
	Siblings	−1.238	0.862	−0.248	−1.437	0.161	1.202			
	Teachers	−0.071	1.656	−0.007	−0.043	0.966	1.076			
Transfer and application	Parents	−8.893	1.180	−0.769	−7.536	0.000 ***	1.134	0.734	0.697	19.982
	Grandparents	−5.643	1.573	−0.356	−3.586	0.001 **	1.076			
	Siblings	−5.476	0.819	−0.702	−6.688	0.000 ***	1.202			
	Teachers5	−4.643	1.573	−0.293	−2.951	0.006 **	1.076			
Problem solving	Parents	−8.929	1.739	−0.711	−5.134	0.000 ***	1.134	0.509	0.441	7.521
	Grandparents	−4.429	2.319	−0.258	−1.910	0.066	1.076			
	Siblings	−4.012	1.207	−0.474	−3.325	0.002 **	1.202			
	Teachers	−2.429	2.319	−0.141	−1.047	0.304	1.076			
Interpersonal interaction	Parents	−6.679	1.956	−0.552	−3.415	0.002 **	1.134	0.333	0.241	3.613
	Grandparents	−4.429	2.608	−0.267	−1.698	0.100	1.076			
	Siblings	−3.012	1.357	−0.369	−2.219	0.034 *	1.202			
	Teachers	−0.429	2.608	−0.026	−0.164	0.871	1.076			
Cognitive quality	Parents	−8.536	1.699	−0.661	−5.023	0.000 ***	1.134	0.557	0.496	9.107
	Grandparents	−7.286	2.266	−0.412	−3.216	0.003 **	1.076			
	Siblings	−4.869	1.179	−0.560	−4.129	0.000 ***	1.202			
	Teachers	−4.786	2.266	−0.271	−2.112	0.043 *	1.076			
	Peers	0								

Notes. * *p* < 0.05, ** *p* < 0.01, *** *p* < 0.001.

**Table 14:** The survey of fathers’ occupation influenced the deep learning ability during play among children with ASD.

Dependent Variable	Model	B	SE	β	t	Sig.	VIF	R^2^	Adjusted R^2^	F
Deep learning ability	Level 1	5.111	12.394	0.049	0.412	0.683	1.025	0.580	0.538	13.790
	Level 2	14.111	8.992	0.190	1.569	0.127	1.046			
	Level 5	28.111	4.391	0.782	6.402	0.000 ***	1.064			
Emotional experience	Level 1	3.833	3.061	0.199	1.252	0.220	1.025	0.262	0.188	3.545
	Level 2	3.333	2.220	0.241	1.501	0.144	1.046			
	Level 5	3.295	1.084	0.492	3.039	0.005 **	1.064			
Problem awareness	Level 1	−1.278	2.346	−0.090	−0.545	0.590	1.025	0.192	0.111	2.376
	Level 2	1.722	1.702	0.170	1.012	0.320	1.046			
	Level 5	2.030	0.831	0.413	2.443	0.021 *	1.064			
Transfer and application	Level 1	2.611	2.276	0.118	1.147	0.260	1.025	0.688	0.657	22.057
	Level 2	1.611	1.651	0.102	0.976	0.337	1.046			
	Level 5	6.534	0.806	0.852	8.104	0.000 ***	1.064			
Problem solving	Level 1	3.222	3.472	0.135	0.928	0.361	1.025	0.384	0.322	6.229
	Level 2	2.722	2.519	0.158	1.081	0.288	1.046			
	Level 5	5.299	1.230	0.637	4.309	0.000 ***	1.064			
Interpersonal interaction	Level 1	−4.444	3.299	−0.193	−1.347	0.188	1.025	0.402	0.342	6.713
	Level 2	3.556	2.394	0.215	1.485	0.148	1.046			
	Level 5	4.632	1.169	0.577	3.963	0.000 ***	1.064			
Cognitive quality	Level 1	1.167	3.140	0.047	0.372	0.713	1.025	0.523	0.476	10.977
	Level 2	1.167	2.278	0.066	0.512	0.612	1.046			
	Level 5	6.321	1.112	0.739	5.682	0.000 ***	1.064			
	Level 3	0								

Notes. * *p* < 0.05, ** *p* < 0.01, *** *p* < 0.001.

## Data Availability

Data are contained within the article, further inquiries can be directed to the corresponding author/s. www.kdocs.cn/l/cpCzdZSqdryo (accessed on 18 October 2025).

## Abbreviations

The following abbreviations are used in this manuscript:

Children with ASD	Children with autism spectrum disorders
Children with TD	Children with typical development
IP	Inclusive play
SP	Solitary play
SEL	Social emotional development
SES	Socioeconomic status
